# Metastatic Esophageal Adenocarcinoma Presenting as Neck Dermal Metastasis

**DOI:** 10.1155/2024/7951391

**Published:** 2024-01-16

**Authors:** Sara Ibrahim-Shaikh, Noah Shaikh, Nour Daboul, Esra Alshaikhnassir, Maria Hafez, Monika E. Freiser

**Affiliations:** ^1^School of Medicine, Indiana University, Indianapolis, Indiana, USA; ^2^Department of Otolaryngology-Head and Neck Surgery, West Virginia University, Morgantown, West Virginia, USA; ^3^Department of Hematology/Oncology, West Virginia University, Morgantown, West Virginia, USA; ^4^Department of Pathology, West Virginia University, Morgantown, West Virginia, USA

## Abstract

Dermal metastasis is a rare manifestation of visceral disease, and esophageal adenocarcinomas represent around only 1% of primaries that present with cutaneous metastasis. In this case, we discuss a patient who presented with a painless submental mass and extensive right neck cutaneous induration and erythema. Core needle biopsy demonstrated poorly differentiated adenocarcinoma. Blood testing also demonstrated elevated carbohydrate antigen 19-9, carcinoembryonic antigen, and alkaline phosphatase. PET/CT followed by esophagoscopy led to the diagnosis of esophageal signet-cell adenocarcinoma primary with isolated dermal metastasis. The patient was started on palliative radiotherapy and passed away two months later from a suspected thoracic fistula and hydropneumothorax.

## 1. Introduction

Cutaneous metastases are rare from systemic malignancy, and the incidence reported is between 0.7% and 9% in various malignancies. Skin metastases usually occur in the advanced stage and are a sign of poor prognosis. They result from lymphatic embolization, hematogenous or contiguous spread, and also by iatrogenic implantation of malignant cells following surgical procedures [1]. Dermal metastasis occurs in 0.8% of cancers and may be the first manifestation of visceral disease [2, 3]. The most common primary sites are breast, lung, colon, melanoma, oral cavity, and ovary [4, 5]. Carcinoma of the esophagus is the sixth most common cause of mortality worldwide. Patients usually present with dysphagia as the main symptom. Esophageal carcinoma most commonly spreads to the lymph nodes, lung, liver, and bones. Cutaneous metastasis can be seen in both variants squamous cell carcinoma and adenocarcinoma [6]. In a review of 401 patients with skin metastasis over 25 years, Wong et al. identified five patients (1.2%) with esophageal cancer primary, all with adenocarcinoma histology. A systematic review of metastatic sites for esophageal cancer demonstrated that less than 7% of esophageal cancers present with skin metastasis [7]. In this case, we discuss a case of esophageal cancer that initially presented as a painless head and neck dermal metastasis.

## 2. Case Presentation

A 71-year-old male with a history of type II diabetes, asthma, gastroesophageal reflux disease, hypertension, and COPD presented to clinic with a two-month history of painless, erythematous submental swelling. Of note, he only had a five-year smoking history, quit 45 years ago, and did not drink. He denied abdominal pain, chronic hiccups, or prior abdominal surgery. He had previously been prescribed a 10-day course of antibiotics with increased size and spread of the swelling. He had additional complaints of the feeling of food sticking without coughing or choking for the past month.

**Figure 1:**
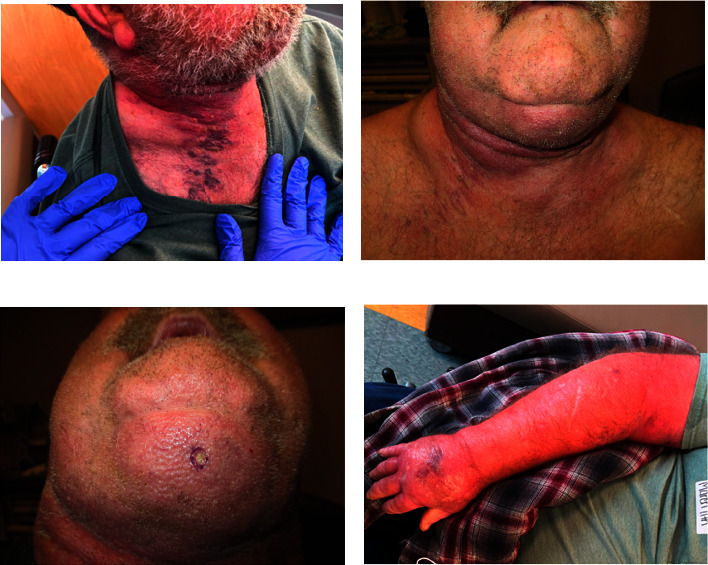
Clinical presentation of the patient. (a) Patient had extensive erythema along his neck. (b, c) Patient had a submental subcutaneous mass that extended to the midline neck. (d) Patient presented with dilated superficial skin venae.

**Figure 2:**
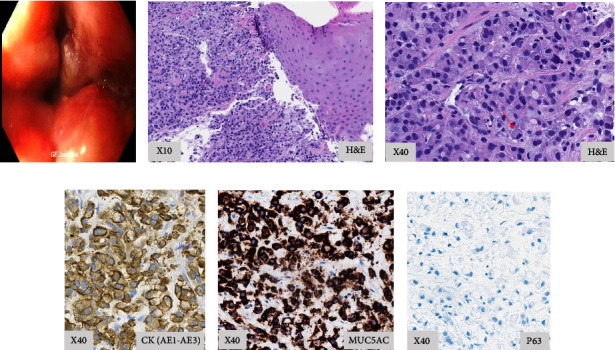
(a) Results from esophagoscopy show a circumferential fungating and ulcerated mass with friable surfaces was found at the gastroesophageal junction. Esophageal biopsy showing poorly differentiated adenocarcinoma, diffuse type. (b) Unremarkable squamous epithelium is shown on the right and the discohesive tumor cells are on the left. (c) Proliferation of signet ring cells (arrow) with intracellular mucin that displaces the nucleus to the side. (d, e) Immunohistochemical staining shows positive staining in highlighting tumor cells in CK (AE1-AE3) and MUC5AC and negative staining of P63, supporting the diagnosis of adenocarcinoma (f).

On examination in clinic, there was extensive erythema along his neck, extending for 26 centimeters in a horizontal direction and 10 centimeters in a vertical direction (Figure 1(a)). He had a very firm, fixed, ill-defined submental subcutaneous mass located midline, involving the mentum to the midline neck measuring 11 × 6 centimeters, nonprotruding but palpable on examination. The erythema and woody feeling of the neck extended another 13 cm right lateral from the main mass (Figures 1(b) and 1(c)). He was noted to have prominent, dilated superficial skin venae and a compressible soft tissue swelling along the right subclavicular area (Figure 1(d)). He had in-office flexible laryngoscopy performed, which showed no masses or lesions of his pharynx or larynx as a source of potential metastatic disease. Esophagoscopy results are demonstrated in Figure 2(a). He underwent a core biopsy of his submental mass during this visit (Figure 2(b)). Pathology demonstrated tumor cells with gland-like structures, signet ring cell morphology (Figure 2(c)). Tumor cells were positive for CK7, MUC5AC (Figures 2(d) and 2(e)), CK20, CDX2, and negative for P63 (Figure 2(f)), CK 5/6, HER2, NKX3.1, p40, PSA, SATB2, SOX10, and TTF-1. There was concern for poorly differentiated adenocarcinoma with suspected origin from pancreatobiliary or upper gastrointestinal tract.

**Figure 3:**
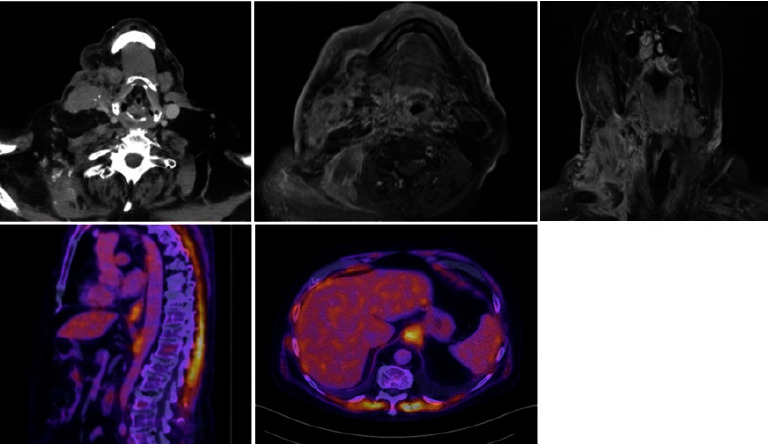
PET/CT scan and MRI of the patient. PET/CT scanning was performed following the intravenous administration of F-18 FDG and intravenous and enteric contrast. F-18 emission and CT attenuation acquisitions were performed, utilizing diagnostic-quality CT. CT FINDINGS: no masses or areas of abnormal enhancement are identified in the head and neck region. No lymphadenopathy is identified. PET FINDINGS: there is hypermetabolic activity in the lower esophagus and GE junction. While this is likely physiologic or inflammatory activity, malignancy is not excluded. MRI results demonstrate extensive expansile infiltrative process representing known metastatic disease involving the right neck and shoulder musculature, particularly the sternocleidomastoid, anterior scalene, and levator scapulae.

He was urgently referred to medical and surgical oncology with positron emission tomography/computed tomography (PET/CT) scan and MRI. PET/CT demonstrated an infiltrative mass in the mid and lower right neck with calcifications in the sternocleidomastoid muscle (SCM) extending caudally to the mid-clavicle and superficially to the skin. There was severe narrowing of the right internal jugular vein (Figure 3). There was PET avidity in the lower esophagus and gastroesophageal (GE) junction and nonspecific hypermetabolic activity in multiple small right hilar and subcarinal lymph nodes with multiple areas of diffuse intramuscular hypermetabolic activity. The thoracic and muscular activities were thought to be due to reactivity or inflammation. MRI scan demonstrated G2 hyperintense, heterogeneously enhancing right neck musculature, including SCM, anterior scalene, and levator scapulae, with extensive fat stranding and extensive dermal thickening.

After a multidisciplinary gastrointestinal (GI) tumor board discussion, he underwent esophagogastroduodenoscopy (EGD) with biopsy, which demonstrated a circumferential mass at the gastroesophageal (GE) junction positive for poorly differentiated adenocarcinoma, signet ring cell type. Bloodwork demonstrated a significantly elevated carbohydrate antigen level of 19-9 (CA 19-9) of 1852 units/mL (normal range: 0–37), elevated carcinoembryonic antigen (CEA) of 10.2 ng/mL (normal range: 0–2.5), and elevated alkaline phosphatase of 212 international units per liter (normal range: 44–147). In this interval period, he developed worsening right upper arm swelling with weeping fluid, which was Doppler negative for deep venous thrombosis (DVT). This swelling was suggested to be secondary to the right-sided supraclavicular mass totally obstructing the right subclavian vein with lymphedema of the right arm.

It was discussed with the patient that he has a stage IV metastatic disease, which was treatable but not curable. He had NextGen Tempus sequencing completed, which demonstrated no microsatellite instability, TP53 p.Y236 stop gain loss of function with 1.7% variant allele fraction, and CTNNB1 p.S37F missense variant gain of function with 0.5% variant allele fraction. PDL-1 testing showed 1-2% positivity of tumor cells.

Starting chemo-immunotherapy with FOLFOX and Nivolumab was discussed with the patient. However, due to symptomatic facial and upper extremity swelling, he was started first on palliative radiation. He started palliative radiotherapy to the right neck with a total dose of 30 Gy. Two months from the patient's initial visit, he was admitted with concerns for hypotension and was found to be in septic shock with a large volume right-sided hydropneumothorax with concerns for paraesophageal space leaking, from which he expired.

## 3. Discussion

Cutaneous metastasis may present as quickly growing dermal or subcutaneous nodules and can mimic dermatitis or cellulitis [8]. Punch or excisional biopsy may be recommended. Complete excision of the dermal metastasis can be performed if it can improve quality of life, pain, and functionality [9]. In this case, we discuss a rare presentation of metastatic signet cell esophageal adenocarcinoma with a large volume dermal metastasis to the neck.

Esophageal cancer is diagnosed in 400,000/year with increasing incidence [10]. There is a predominance of adenocarcinoma histology in the Western world [11]. 50–64% of patients present with inoperable disease [12, 13], with the majority of patients with inoperable disease dying within 21 months [13]. This patient was not seen in a specialty clinic until two months after he first noted the neck mass. Delay was in part patient-related, in part due to time spent on a trial of antibiotics, and in part due to scheduling into the specialty clinic as a routine rather than urgent consult. Biopsy was performed on first presentation to our clinic and he started palliative radiation, ultimately expiring two months after his initial visit with us.

A systematic review of esophageal cancer metastases to unexpected sites found that the most common unusual metastases site for esophageal adenocarcinoma is to the skin [6]. The skin lesions may be observed in different parts of the human body. Most commonly this would be in the scalp [14], and head and neck locations were more common than abdominal, pelvic, thoracic, extremity or other skin locations. Only six cases of esophageal adenocarcinoma were reported to metastasize to multiple skin and muscle sites. Our patient demonstrated extensive neck, skin, and muscle metastasis. Metastatic skin lesions are often asymptomatic. However, their physical examination may reveal inflammatory rashes, papules or patches, alopecia, neoplasia, erythematous, indurated plaques or skin nodules [15]. It is important for physicians evaluating skin lesions to be aware that esophageal carcinoma can present with extensive painless induration and erythema, with biopsy indicated the same day. The result of poorly differentiated adenocarcinoma should prompt initiation of tumor marker blood work and PET/CT imaging to find the tumor primary site, referral to oncologic medical and surgical teams, and timely tumor board discussion for this rare presentation. Patients with skin metastatic disease usually have significantly poorer prognosis with reported survival rates of <1 year after the identification of metastatic lesions. The treatment is usually aimed to palliation through possible resection with chemotherapy and radiotherapy [16].

## Data Availability

All data generated or analyzed in this case report are included in this article.
